# Cabozantinib real‐world effectiveness in the first‐through fourth‐line settings for the treatment of metastatic renal cell carcinoma: Results from the International Metastatic Renal Cell Carcinoma Database Consortium

**DOI:** 10.1002/cam4.3717

**Published:** 2021-01-18

**Authors:** Chun Loo Gan, Shaan Dudani, J. Connor Wells, Frede Donskov, Sumanta K. Pal, Nazli Dizman, Nityam Rathi, Benoit Beuselinck, Flora Yan, Aly‐Khan A. Lalani, Aaron Hansen, Bernadett Szabados, Guillermo de Velasco, Ben Tran, Jae Lyun Lee, Ulka N. Vaishampayan, Georg A. Bjarnason, Mathushan Subasri, Toni K. Choueiri, Daniel Y. C. Heng

**Affiliations:** ^1^ Tom Baker Cancer Centre University of Calgary Calgary AB Canada; ^2^ Aarhus University Hospital Aarhus Denmark; ^3^ City of Hope Comprehensive Cancer Center Duarte CA USA; ^4^ Huntsman Cancer Hospital Salt Lake City UT USA; ^5^ University Hospitals Leuven Leuven Cancer Institute Leuven Belgium; ^6^ University of Texas Southwestern Medical Center Dallas TX USA; ^7^ Juravinski Cancer Centre McMaster University Hamilton ON Canada; ^8^ Princess Margaret Cancer Centre Toronto ON Canada; ^9^ Queen Mary University of London Barts Cancer Institute London UK; ^10^ University Hospital 12 de Octubre Madrid Spain; ^11^ Personalised Oncology Division Walter and Eliza Hall Institute of Medical Research Parkville Vic Australia; ^12^ Peter MacCallum Cancer Center Parkville Vic Australia; ^13^ Asan Medical Center University of Ulsan College of Medicine Seoul South Korea; ^14^ Barbara Ann Karmanos Cancer Institute Detroit MI USA; ^15^ Sunnybrook Odette Cancer Centre Toronto ON Canada; ^16^ London Ontario Health Sciences Centre London ON Canada; ^17^ Dana‐Farber Cancer Institute/Brigham and Women's Hospital/Harvard Medical School Boston MA USA

**Keywords:** cabozantinib, first line, fourth line, IMDC, real‐world, renal‐cell carcinoma, response rates, second line, survival, third line

## Abstract

**Background:**

Cabozantinib is approved for metastatic renal cell carcinoma (mRCC) based on the METEOR and CABOSUN trials. However, real‐world effectiveness and dosing patterns of cabozantinib are not well characterized.

**Methods:**

Patients with mRCC treated with cabozantinib between 2011 and 2019 were identified and stratified using the International mRCC Database Consortium (IMDC) risk groups. First‐ (1L), second‐ (2L), third‐ (3L), and fourth‐line (4L) overall response rate (ORR), time to treatment failure (TTF), and overall survival (OS) were analyzed. Dose reduction rates and their association with TTF and OS were determined.

**Results:**

A total of 413 patients were identified. The ORRs across 1L to 4L were 32%, 26%, 25%, and 29%, respectively, and the median TTF rates were 8.3, 7.3, 7.0, and 8.0 months, respectively. The median OS (mOS) rates in 1L to 4L were 30.7, 17.8, 12.6, and 14.9 months, respectively. For patients treated with 1L PD(L)1 combination agent (*n* = 31), 2L cabozantinib had ORR of 22%, median TTF of 5.4 months, and mOS of 17.4 months. About 50% (129/258) of patients required dose reductions. The TTF and mOS were significantly longer for patients who required dose reduction vs. patients who did not, with an adjusted hazard ratio of 0.37 (95% CI 0.202–0.672, *p* < 0.01) and 0.46 (95% CI 0.215–0.980, *p* = 0.04), respectively. Limitations include the retrospective study design and the lack of central radiology review.

**Conclusion:**

The ORR and TTF of cabozantinib were maintained from the 1L to 4L settings. Dose reductions due to toxicity were associated with improved TTF and OS. Cabozantinib has clinical activity after 1L Immuno‐oncology combination agents.

## Graphical abstract

Cabozantinib demonstrates activity for use in real‐world patients with metastatic renal cell carcinoma across the first‐ to fourth‐line treatment settings.

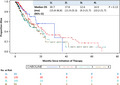

## 1 INTRODUCTION

The sequencing of therapies in metastatic renal cell carcinoma (mRCC) has become increasingly topical and relevant. Examining practice patterns and clinical effectiveness of drugs in real‐world populations is important.

Cabozantinib is an oral tyrosine kinase inhibitor (TKI) that targets vascular endothelial growth factor receptor (VEGFR), MET, and AXL.^1^ The randomized phase III METEOR study demonstrated that cabozantinib has superior progression free survival (PFS), overall survival (OS), and overall response rate (ORR) compared to everolimus in patients who progressed after previous VEGFR‐targeted therapy, leading to its approval by the U.S. Food and Drug Administration in 2016.^2^ In the following year, cabozantinib was further approved for use in the first‐line setting for patients with IMDC intermediate‐/poor‐risk disease. This was based on the randomized phase II CABOSUN trial, which demonstrated a significant improvement in PFS and ORR over the then standard‐of‐care sunitinib for IMDC intermediate‐/poor‐risk patients.^3^

Practice patterns and real‐world effectiveness of cabozantinib are not well characterized over different lines of therapy. With the recent publication of major phase III studies showing superiority of first‐line immuno‐oncology (IO) combinations (such as ipilimumab + nivolumab, axitinib + pembrolizumab, or axitinib + avelumab) vs. sunitinib,^4^, ^5^, ^6^ documenting the effectiveness of drugs such as cabozantinib is informative as clinical trials may not be available in these settings.

Furthermore, the need for dose reduction or titration according to toxicities in TKIs such as sunitinib, pazopanib, and axitinib is associated with improved outcomes,^7^, ^8^ but this remains to be studied with cabozantinib. It may be that dose reduction due to toxicity is a surrogate for adequate drug exposure and is associated with improved outcomes.^7^, ^9^

We conducted a multicenter retrospective study in patients with mRCC treated with cabozantinib across the first‐line (1L), second‐line (2L), third‐line (3L), fourth‐line (4L), and post 1L immuno‐oncology (IO) combination settings.

## 2 PATIENTS AND METHODS

### 2.1 Study design and patient selection

A retrospective analysis was conducted using the IMDC database, which included data from 38 international centers involving 10,200 consecutive patients with mRCC. Data were collected from hospital and pharmacy records between 1 January 2011 and 16 September 2019, using uniform database software and templates. Institutional review board approval was obtained from each participating center.

All patients with clear cell or non‐clear cell mRCC treated with cabozantinib in the 1L, 2L, 3L, and 4L cabozantinib settings were identified. Patients who received cabozantinib as part of previously reported clinical trials were eligible for inclusion.

### 2.2 Outcome measurement

The primary endpoints of this study were ORR, time to treatment failure (TTF), and OS. ORR was investigator assessed and reported in all evaluable patients. The best overall response was documented as complete response (CR), partial response (PR), stable disease (SD), or progressive disease (PD) as per Response Evaluation Criteria in Solid Tumors (RECIST) version 1.1 guidelines, where available.^10^ The ORR included the percentage of patients with CR and PR as their best response. TTF was defined as the time from the initiation of systemic therapy to treatment discontinuation for any reason or censored at the time of the last follow‐up. OS was calculated from the time of initiation of first‐line systemic therapy to death from any cause or censored at the time of the last follow‐up.

Information in relation to dosing patterns such as the need for dose reduction due to toxicity, median average daily dose, and the treatment discontinuation rate due to toxicity was collected. The ORR, TTF, and OS of patients who required dose reduction vs. not were determined.

### 2.3 Statistical analysis

Patient demographics and baseline characteristics were described using frequencies and percentages (%) for categorical variables, and medians and interquartile ranges for continuous variables. TTF and OS were calculated using the Kaplan and Meier method. Patients in the 2L and 3L settings were stratified by IMDC criteria and compared for OS because these data were sufficiently powered to perform such an analysis.

A multivariable Cox regression analysis was performed in the 1L to 4L therapy settings, examining the need for dose reduction vs. not in relation to OS with the use of adjusted hazard ratios (HRs) to control for imbalances in IMDC risk factors (corrected calcium greater than the upper limit of normal, neutrophils greater than the upper limit of normal, platelets greater than the upper limit of normal, hemoglobin less than the lower limit of normal, Karnofsky performance status <80%, and time from diagnosis to treatment <1 year). Patients with 0 factors vs. 1–2 factors vs. 3 or more factors are deemed favorable‐, intermediate‐, and poor‐risk, respectively.^11^

The case deletion method was used when missing data were encountered. SAS statistical software (version 9.4; SAS Institute Inc.) was used to perform statistical analyses.

## 3 RESULTS

### 3.1 Patients characteristics

A total of 413 patients who received cabozantinib in the 1L, 2L, 3L, and 4L settings were identified from the IMDC database, and their median follow‐up was 14.7, 14.5, 10.0, and 10.0 months, respectively. Of the 320 patients who had histological subtyping available, 78% (248/320) had ccRCC and 22% (72/320) had non‐clear cell mRCC. The median age at the time of starting cabozantinib was 64 years. Overall, 71% of patients had a Karnofsky performance status score of ≥80, and 83% had prior nephrectomy. The majority of the patients (89%) were of IMDC intermediate‐/poor‐risk. Detailed baseline characteristics of the patients are summarized in Table 1.

**TABLE 1:** Baseline Characteristics, IMDC risk factors, and prior treatment

	First line (*N* = 34)	Second line (*N* = 143)	Third line (*N* = 142)	Fourth line (*N* = 94)
Age, median (IQR) (at diagnosis)	62 (57–70)	57 (49–64)	56 (50–63)	57 (50–64)
Age, median (IQR) (at starting cabozantinib)	65 (57–76)	61 (53–67)	62 (54–69)	65 (56–71)
Male	88% (30/34)	80% (115/143)	77% (109/142)	78% (73/94)
Clear cell histology	44% (10/23)	82% (85/104)	75% (88/118)	87% (65/75)
Sarcomatoid histology	14% (3/21)	20% (20/98)	19% (21/109)	6% (4/71)
Prior nephrectomy	74% (25/34)	76% (109/143)	87% (124/142)	88% (83/94)
Liver metastases	9% (3/34)	17% (24/143)	18% (25/143)	16% (15/94)
Bone metastases	27% (9/34)	39% (55/143)	30% (43/143)	26% (24/94)
Lung metastases	59% (20/34)	67% (96/143)	70% (100/143)	69% (65/94)
Brain metastases	6% (2/34)	4% (5/143)	7% (10/143)	2% (2/94)
IMDC risk groups				
Favorable	14% (4/29)	12% (13/107)	11% (11/98)	9% (6/69)
Intermediate	41% (12/29)	64% (68/107)	59% (58/98)	49% (34/69)
Poor	45% (13/29)	24% (26/107)	30% (29/98)	42% (29/69)
IMDC risk factors at time of initiation of cabozantinib				
KPS < 80%	29% (9/31)	21% (25/122)	31% (40/129)	40% (35/88)
Diagnosis to therapy <1 year	70% (23/33)	73% (105/143)	51% (73/142)	49% (46/94)
Calcium > ULN	7% (2/31)	6% (7/122)	13% (14/107)	20% (14/71)
Hemoglobin < LLN	58% (18/31)	59% (76/130)	65% (82/126)	70% (59/84)
Neutrophils > ULN	19% (6/31)	12% (15/129)	23% (27/120)	23% (18/80)
Platelets >ULN	16% (5/31)	8% (10/130)	18% (22/124)	24% (20/83)
Immediate prior therapy				
TKI		73% (104/143)	32% (45/139)	28% (25/89)
Everolimus + Lenvatinib		1% (2/143)	2% (3/139)	1% (1/89)
Single agent IO		1% (2/143)	57% (79/139)	55% (49/89)
IOIO		7% (10/143)	5% (7/139)	8% (7/89)
IOVE		15% (21/143)	1% (2/139)	2% (2/89)
Other (everolimus, temsirolimus, IL2, interferon)		3% (2/143)	2% (3/139)	6% (5/89)
Prior IO any line		23% (33/143)	75% (107/142)	88% (83/94)
Single agent IO		1% (2/143)	61% (87/142)	77% (72/94)
IOIO		7% (10/143)	6% (8/142)	11% (10/94)
IOVE		15% (21/143)	8% (12/142)	1% (1/94)

Abbreviations: IOIO, immuno‐oncology agent combinations; IOVE, immuno‐oncology + VEGF inhibitor combinations; KPS, Karnofsky performance status; LLN, lower limit of normal; ULN, upper limit of normal.

### 3.2 ORR, TTF, and OS for 1L–4L treatment

**FIGURE 1:**
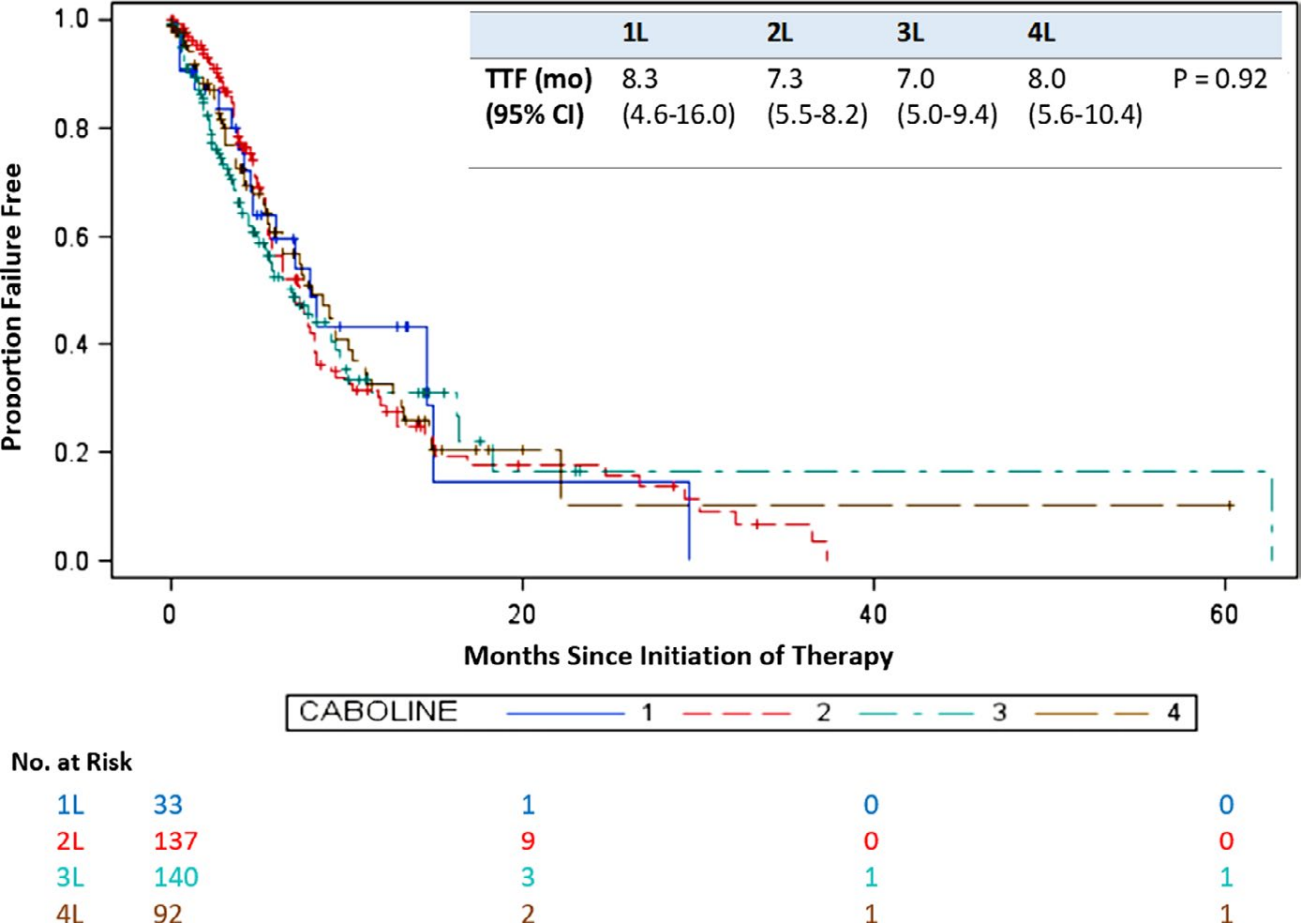
Time to treatment failure for first‐ to fourth‐line cabozantinib. 1L, first line; 2L, second line; 3L, third line; 4L, fourth line; CI, confidence interval; TTF, time to treatment failure

**FIGURE 2:**
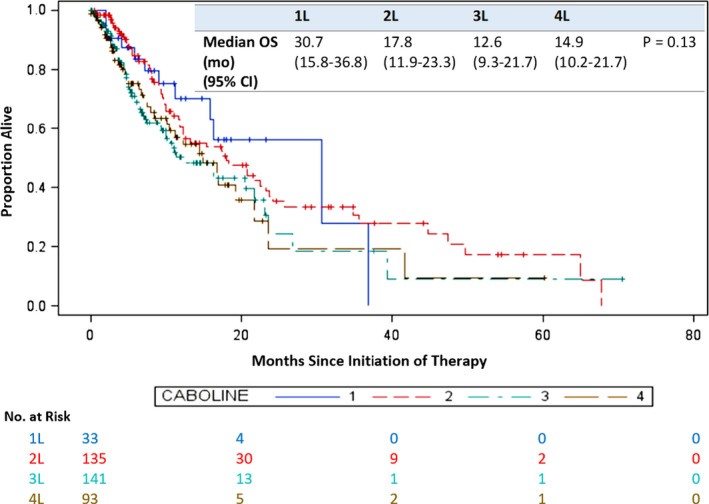
Overall survival for first‐ to fourth‐line cabozantinib. 1L, first line; 2L, second line; 3L, third line; 4L, fourth line; CI, confidence interval; OS, overall survival

The ORR, TTF, and OS of patients treated with cabozantinib in the 1L–4L of therapy were comparable (Table 2; Figures 1 and 2) except mOS after 1L, which was much longer because these patients were therapy naïve. For patients treated with 1L PD(L)1 combination (*n* = 31), 2L cabozantinib had ORR of 22%, median TTF of 5.4 months, and median OS (mOS) of 17.4 months.

**TABLE 2:** First‐line to fourth‐line cabozantinib treatment outcomes

	First line	Second line	Third line	Fourth line		Post first‐line IO Combos
ORR	32% (9/28)	26% (28/109)	25% (25/102)	29% (19/65)		22% (5/23)
Best response						
CR	0% (0/28)	1% (1/109)	0% (0/102)	2% (1/65)	0% (0/23)	
PR	32% (9/28)	25% (27/109)	25% (25/102)	28% (18/65)	22% (5/23)	
SD	50% (14/28)	52% (57/109)	48% (49/102)	49% (32/65)	57% (13/23)	
PD	18% (5/28)	22% (24/109)	28% (28/102)	22% (14/65)	21% (5/23)	
TTF (mo) (95% CI)	8.3 (4.6–16.0)	7.3 (5.5–8.2)	7.0 (5.0–9.4)	8.0 (5.6–10.4)	5.4 (4.4–5.8)	
Median OS (mo) (95% CI)	30.7 (15.8–36.8)	17.8 (11.9–23.3)	12.6 (9.3–21.7)	14.9 (10.2–21.7)	17.4 (4.8–23.3)	

Note

IO Combos include Ipilimumab/Nivolumab (*n* = 5) and various PD(L)1 + VEGF inhibitor combinations (*n* = 18).

Abbreviations: CI, confidence interval; CR, complete response; PD, progressive disease; PR, partial response; SD, stable disease.

Table 3 describes the mOS in patients who are favorable, intermediate‐, and poor‐risk by IMDC criteria in the 2L and 3L settings.

**TABLE 3:** IMDC risk group and median overall survival (OS)

IMDC risk group	Second‐line median OS in months (*p* < 0.01)	Third‐line median OS in months (*p* < 0.01)
Favorable	34.8 (5.52–NR)	31.5 (23.6–39.3)
Intermediate	18.0 (12.3–35.6)	20.5 (10.1–21.8)
Poor	9.8 (7.4–20.8)	6.9 (4.1–10.9)

### 3.3 Dose reduction and treatment outcomes

**FIGURE 3:**
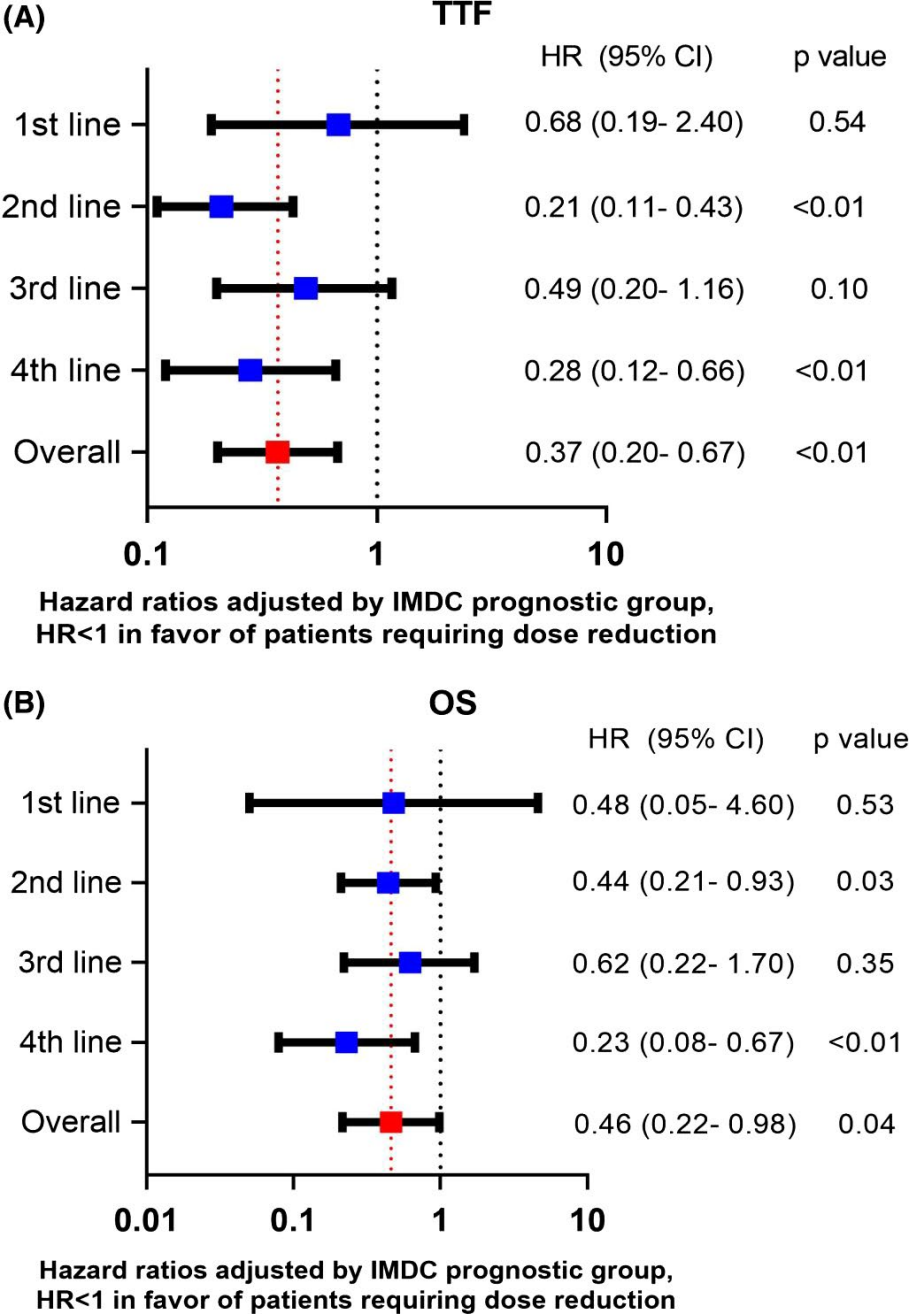
(A, B) Effect of the need for dose reduction due to toxicity on time to treatment failure (TTF) and overall survival (OS)

Of the 413 patients, 258 had evaluable data for dose reduction. Overall, 50% (129/258) of patients required dose reductions. The median time to dose reduction was 1.2 months (95% CI 0.91–1.57). Across all lines of therapy, the TTF and mOS were significantly longer for patients who required dose reduction vs. patients who did not, with an adjusted HR of 0.37 (95% CI 0.202–0.672, *p* < 0.01) and 0.46 (95% CI 0.215–0.980, *p* = 0.04), respectively (Figure 3A,B). The ORR did not differ in patients with or without dose reduction (23% vs. 26%, *p* = 0.69; Table 4). However, there were fewer patients with PD as the best response in the dose reduction group vs. not (32% vs. 16%, *p* < 0.01).

**TABLE 4:** ORR of patients with or without dose reduction

	Patients with dose reduction (*n* = 108)	Patients without dose reduction (*n* = 98)	*p*‐value
ORR	23% (25)	26% (25)	0.69
CR	1% (1)	1% (1)	
PR	22% (24)	25% (24)	
SD	61% (66)	43% (42)	
PD	16% (17)	31% (31)	

The average daily dose of cabozantinib was 36.6, 37.8, 34.8, and 34.7 mg for 1L to 4L settings, respectively. The dose patterns are summarized in Table 5.

**TABLE 5:** Dose patterns of patients across first‐line to fourth‐line cabozantinib

	1L	2L	3L	4L
Number of patients who required dose reduction (%, *n*/*n*)	42 (10/24)	58 (56/96)	37 (33/89)	56 (31/55)
Median final daily dose for patients who required dose reduction	40 mg	40 mg	40 mg	40 mg
Average daily dose	36.6 mg	37.8 mg	34.8 mg	34.7 mg
Percentage of patients discontinued treatment due to toxicities (%, *n*/*n*)	32 (7/22)	16 (14/85)	26 (17/65)	30 (11/37)

## 4 DISCUSSION

Our study describes the efficacy and treatment outcomes of cabozantinib in a real‐world mRCC patient population. We demonstrated that cabozantinib has clinical activity across 1L to 4L for mRCC. Notably, the ORR and TTF did not deteriorate from increasing lines of prior therapies, suggesting cabozantinib is an active drug regardless of the line of therapy in which it is used. Although the number of patients in the 1L setting is small (*n* = 34), the ORR rate of 32% was consistent with the investigator‐assessed ORR of 33% reported in CABOSUN study.^3^ The ORR across 2L–4L (26%, 25%, and 29%, respectively) in our study appears to be higher than the independent radiology reviewed ORR of 17% reported in the METEOR study.^2^ This may in part be due to the lack of central radiology review for response assessment for our patients.

Other real‐world experiences of cabozantinib exist in the literature. In the Polish retrospective managed access program (MAP) of 115 patients treated with cabozantinib in the 2L setting and beyond, the ORR was 19%, and the median PFS was 12.5 months (95% CI, 9.2–14.2 months).^12^ Similarly, in an Italian MAP study of 96 patients who received cabozantinib in 2L setting and beyond, the ORR was 36%, median PFS was 8.0 months (95% CI, 0.5–10.8 months), and mOS was not reached.^13^ Furthermore, a study from the U.K. expanded access program for cabozantinib (*n* = 128) showed comparable results with ORR of 26%, median PFS of 7.7 months (95% CI 5.3–10.1), and mOS of 9.1 months (95% CI 6.6–11.6) in patients who received at least one prior line of therapy.^14^ These studies provided further evidence of the efficacy of cabozantinib use in a real‐world population.

The approval of combination IOIO (ipilimumab and nivolumab) and IO + VEGF (IOVE) inhibitors (pembrolizumab/axitinib and axitinib/avelumab) as first‐line treatment options make the sequencing of therapies increasingly debatable and poses a challenge for clinicians.^4^, ^5^, ^6^ This study benchmarks cabozantinib outcomes after these therapies, which are important for reimbursement and regulatory agencies. In our study, in patients treated with 1L IOIO (*n* = 5) or IOVE (*n* = 18), 2L cabozantinib had an ORR of 22%, a median TTF of 5.4 months, and a mOS of 17.4 months, suggesting that cabozantinib is active as a 2L therapy after prior IOIO or IOVE therapies. However, the ORR and TTF observed here appear to be lower than the ORR and TTF found in patients who received cabozantinib across 1L–4L settings. This could be related to a much smaller sample size in the post IO 2L cabozantinib setting. Another possible explanation is that only five out of the 23 patients were VEGFR‐inhibitor naïve. The sample size of 23 patients would preclude any meaningful comparison for cabozantinib efficacy post 1L IOIO vs. IOVE. A retrospective study presented at the ESMO 2018 involving 86 patients who received cabozantinib after progression on prior IO therapy reported an ORR of 36% and a median TTF of 6.5 months (95% CI 5.3–8.1).^15^ In this study, the number of evaluable patients who received IOVE was only seven, limiting the interpretation of the reported ORR and TTF in this context. The median number of prior therapies in this study was two. In a study of 84 patients who received prior checkpoint inhibitors, Iacovelli and colleague reported an impressive ORR of 52% and PFS of 11.5 months (95% CI 8.3–14.7) with the use of cabozantinib in the 3L and beyond setting.^16^ These studies suggest that cabozantinib is active in the post IO setting. Further prospective study is warranted to investigate the efficacy of TKIs such as cabozantinib in the post first‐line IO combination treatment setting. On the same note, sequencing of cabozantinib in the second line and beyond setting was examined in a recent study, where it was found that patients with IMDC good‐risk disease experienced significantly longer time‐to‐strategy failure and OS with 2L nivolumab followed by 3L cabozantinib (*n* = 89), compared to 2L cabozantinib and subsequent 3L Nivolumab (*n* = 29).^17^ This finding will require validation in future prospective studies.

Cabozantinib may cause treatment‐limiting adverse events, necessitating the need for dose reduction. Our study showed that 50% of patients required dose reduction, and the median daily dose was 40 mg. The dose reduction rate is consistent with data from the pivotal METEOR and CABOSUN trials, where 71% and 67% of the patients experienced grade 3–4 adverse events, respectively; while the dose reduction rates were 64% and 46%,^3^, ^18^ respectively, resulting in a median average daily dose of 42.8 and 50.3 mg, respectively. The lower median daily dose in our study may reflect the real‐world population, where patients are typically less robust and often have poorer performance status and increased medical comorbidities compared to clinical trials population.

Importantly, patients who required dose reductions consistently experienced numerically longer OS and TTF across 1L to 4L of cabozantinib treatment. This observation is consistent with other data where dose reductions of VEGFR‐TKIs mandated by toxicities experienced with sunitinib or pazopanib may be associated with better survival.^7^, ^19^, ^20^ Grade 3–4 clinical TKI‐related toxicities were associated with significant improvement of OS in a study of 54 patients receiving sunitinib or sorafenib.^19^ In addition, sunitinib‐associated hypertension and higher diastolic blood pressure in patients receiving axitinib were shown to be associated with improved treatment response and OS.^21^, ^22^ Furthermore, a post hoc analysis of the COMPARZ trial found significantly improved median ORR, PFS, and OS for patients who required dose reductions for pazopanib.^7^ Thus, patients who require dose reductions seem to have improved outcomes compared to those who do not require dose reductions.^9^ This observation does not mean that we should dose reduce all patients, but instead, it suggests that toxicity could be a surrogate marker for sufficient drug exposure and is a biomarker for efficacy. Therefore, the efficacy of cabozantinib might be improved with individualized dosing based on toxicity as has been studied for sunitinib and axitinib in phase II trials.^23^, ^24^ This requires prospective evaluation with cabozantinib.

The limitations of this study include its retrospective nature and potential selection bias. These are mitigated by the use of continuous patient series. There are several potential biases that may contribute to the longer mOS and TTF seen in our patients who required dose reductions. Anecdotally, many patients who are on treatment for a long enough period of time will eventually require dose reductions. Therefore, patients who required dose reductions may simply have longer exposure to the drug and likely are the responders hence inducing a guarantee‐time bias.^25^ However, the median time to dose reduction was 1.2 months, which was much shorter than the TTF, possibly reducing the influence of guarantee‐time bias. Due to the retrospective nature of our study, there is incomplete data regarding dose schedules, drug toxicities, and the timing and level of dose reduction. Further prospective studies to examine alternate schedules for cabozantinib is warranted for dose individualization.

## 5 CONCLUSIONS

To our knowledge, our study is the largest real‐world analysis of the use of cabozantinib in patients with mRCC. The ORR and TTF of cabozantinib were maintained from the 1L to 4L therapy settings. The need for dose reduction due to toxicity was associated with improved TTF and OS. This contributes to mounting evidence that TKI toxicity requiring dose reduction is associated with better outcomes and that toxicity is a surrogate of adequate drug exposure. In the 2L and 3L settings, the IMDC criteria appropriately stratified patients into favorable, intermediate, and poor‐risk groups for OS. Cabozantinib has clinical activity after first‐line IO combination therapies.

## ETHICS APPROVAL

This project has received ethics approval from the Health Research Ethics Board of Alberta—Cancer Committee: HREBA.CC‐17‐0458_REN3 which expires 19 May 2021.

## CONFLICT OF INTEREST

Dr. Well reports receiving travel and accommodation support from Pfizer; Dr. Donskov reports institutional research funding from Pfizer and Ipsen; Dr. Pal reports receiving fees for consulting and serving on advisory roles from Pfizer, Novartis, Aveo, Myriad Pharmaceuticals, Genentech, Exelixis, Bristol‐Myers Squibb, Astellas Pharma, Ipsen and Eisai, and honoraria from Novartis, Medivation and Astellas Pharma, and institutional research funding from Medivation; Dr. Beuselinck reports receiving fee from Pfizer for speakers' bureau and travel and accommodations support from Pfizer; Dr. Lalani reports receiving fees for consulting and serving on advisory role from Eisai, Merck, Ipsen, Pfizer, Roche/Genentech, Bristol‐Myers Squibb, Abbvie, Janssen, Tersera, Bayer and Astellas Pharma, and Honoraria from Pfizer, Roche/Genentech, Merck, Novartis, Astellas Pharma, Bayer, Tersera, Bristol‐Myers Squibb, and institutional research funding from Bristol‐Myers Squibb, Novartis, Roche and Ipsen; Dr. Hansen reports receiving fees for consulting and serving on advisory role from Genentech/Roche, Merck, GlaxoSmithKline, Bristol‐Myers Squibb, Novartis, Boston Biomedical and Boehringer Ingelheim, and receiving Honoraria from Merck, AstraZeneca/MedImmune, GlaxoSmithKline/Novartis and Bristol‐Myers Squibb, and institutional research funding from Karyopharm Therapeutics, Merck, Bristol‐Myers Squibb, Boehringer Ingelheim, GlaxoSmithKline, Novartis, Roche/Genentech and Janssen; Dr. Szabados reports receiving travel and accommodation support from Roche/Genentech, and Honoraria from Merck; Dr. Velasco reports receiving fees for consulting and serving on advisory roles from Janssen, Pfizer, Novartis, Bayer, Astellas Medivation, Bristol‐Myers Squibb, Ipsen and MSD, and receiving Honoraria from Pfizer and Ipsen, institutional research funding from Ipsen; Dr. Tran reports receiving fees for consulting and serving on advisory roles from Amgen, Astellas Pharma, Bayer, Sanofi, Tolmar, Janssen‐Cilag, Bristol Myers Squibb, Ipsen and MSD oncology, and travel and accommodation support from Amgen and Astellas Pharma, and Honoraria from Astellas Pharma, Janssen‐Cilag, Sanofi, Tolmar and Amgen, and institutional research funding from Astellas Pharma, Janssen‐Cilag, Amgen, Pfizer, Genentech, AstraZeneca, Bayer, Pfizer, Janssen‐Cilag, Astellas Pharma, Bristol‐Myers Squibb, Merck Sharp and Dohme; Dr. Lee reports receiving fees for consulting and serving on advisory boards from Pfizer, Sanofi/Aventis, Bristol Myers Squibb and Alteogen, and Honoraria from Bristol Myers Squibb, AMgen, Astellas Pharma, Pfizer, and institutional research funding from Pfizer, Janssen, Novartis, Bristol Myers Squibb, Roche/Genentech, AstraZeneca/Medimmune, MSD, Bayer Schering Pharma and Seattle Genetics; Dr. Vaishampayan reports receiving fees for consulting and serving on advisory roles from Pfizer, Bristol Myers Squibb, Exelixis, Bayer and EMD Serono, and speakers’ bureau from Pfizer, Bayer, Bristol Myers Squibb, Exelixis, Sanofi and Eisai, and Honoraria from Pfizer, Bayer, Sanofi, Bristol Myers Squibb and Exelixis, and institutional research funding from Astellas Pharma, Exelixis, Pfizer and Bristol Myers Squibb; Dr. Bjarnason reports receiving fees for consulting and serving on advisory roles from Pfizer, Novartis, Bristol Myers Squibb, Eisai and Ipsen, and travel and accommodations support from Pfizer and Novartis and Honoraria from Pfizer, Novartis, Bristol Myers Squibb, Eisai and Ipsen, and institutional research funding from Pfizer and Merck; Dr. Choueiri reports receiving fees for consulting and serving on advisory roles from Pfizer, Bayer, Novartis, GlaxoSmithKline, Merck, Bristol Myers Squibb, Roche/Genentech, Eisai, Foundation Medicine, Cerulean Pharma, AstraZeneca, Prometheus Laboratories, Alligent, Ipsen, Corvus Pharmaceuticals, Lpath, Alexion Pharmaceuticals, Sanofi/Aventis, Peloton Therapeutics, UpToDate, NCCN, Michael J. Hennessy Associates, Analysis Group, Kidney cancer Association, Clinical Care options, PlatformQ Health, Navinata Health, Harborside Press, ASCO, The New England Journal of Medicine, Lancet Oncology, EMD Serono, HERON, Lilly and ESMO, and holding leadership positions at Dana Farber Cancer hospital, NCCN, Kidney cancer Association, KidneyCan and ASCO, and receiving medical writing and editorial assistance support that may have been funded by Communications companies funded by pharmaceutical companies such as ClinicalThinking, Health Interactions, Envision Pharma Group, Fishawack Group of Companies and Parexel, stock and other ownership interests from Pionyr and Tempest Therapeutics, and receiving Honoraria from NCCN, UpToDate, Michael J. Hennessy Associates, ASCO, Harborside Press, Analysis Group, AstraZeneca, Alexion Pharmaceuticals, Sanofi/Aventis, Bayer, Bristol‐Myers Squibb, Genentech/Roche, GlaxoSmithKline, Merck, Novartis, Peloton Therapeutics, Pfizer, Corvus Pharmaceuticals, Ipsen, Foundation Medicine, Eisai, PlatformQ Health, Clinical Care options, Navinata Health, Kidney cancer association, exelixis, prometheus, Lpath—The New England Journal of Medicine, Lancet Oncology, Cerulean Pharma, Alligent, EMD Serono, Heron and Lilly, and institutional research funding from Pfizer, Novartis, Merck, Exelixis, Tracon Pharma, GlaxoSmithKline, Bristol Myers Squibb, AstraZeneca, Peloton Therapeutics, Roche/Genentech, Celldex, Agensys, Eisai, Takeda, Prometheus, Ipsen, Corvus Pharmaceuticals, Cerulean Pharma, Seattle Genetics/Astellas, Bayer, Foundation Medicine, Roche, Calithera Biosciences, Analysis Group, NCI, CDMRP/DOD and GATEWAY for Cancer Research; Dr. Heng reports receiving fees for consulting and serving on advisory roles from Pfizer, Novartis, Bristol Myers Squibb, Janssen, Astellas Pharma, Ipsen, Eisai and Merck, and institutional research funding from Pfizer, Novartis, Exelixis, Bristol Myers Squibb and Ipsen. The other authors made no disclosures.

## AUTHORS CONTRIBUTIONS

Conception and design: Gan, Heng; Acquisition of data: Gan, Dudani, Wells, Donskov, Pal, Dizman, Rathi, Beuselinck, Yan, Lalani, Hansen, Szabados, de Velasco, Tran, Lee, Vaishampayan, Bjarnason, Subasri, Choueiri, Heng; Analysis and interpretation of data: Gan, Heng; Drafting of the manuscript: Gan, Heng; Critical revision of the manuscript for important intellectual content: Gan, Dudani, Wells, Donskov, Pal, Dizman, Rathi, Beuselinck, Yan, Lalani, Hansen, Szabados, de Velasco, Tran, Lee, Vaishampayan, Bjarnason, Subasri, Choueiri, Heng; Statistical analysis: Gan, Heng; Obtaining funding: None; Administrative, technical, or material support: None; Supervision: Heng; Other (specify): None.

## Data Availability

The data that support the findings of this study are available on request from the corresponding author. The data are not publicly available due to privacy or ethical restrictions.
